# Flagellin FLiS improves the resistance of cotton to Verticillium wilt through the signaling pathways of salicylic acid and jasmonic acid

**DOI:** 10.3389/fpls.2025.1595529

**Published:** 2025-06-03

**Authors:** Yujing Liu

**Affiliations:** School of Advanced Manufacturing, Fuzhou University, Jinjiang, China

**Keywords:** Flagellin S, upland cotton, immune response, Verticillium wilt, disease resistance

## Abstract

Verticillium wilt of cotton is a soil-borne vascular bundle disease. There is still a lack of effective methods for controlling and preventing Verticillium wilt of cotton. There are few reports on the research of the mechanism by which flagellin S (FLiS) protein induces cotton immunity. The mechanism by which the FLiS protein induces immune responses in cotton was analyzed through prokaryotic expression and purification, physiological and biochemical techniques, and qRT-PCR (quantitative real-time polymerase chain reaction) technology. The purpose of this study was to determine the role and mechanism of FLiS in improving the resistance of cotton to Verticillium wilt. An endophytic bacterium (Pseudomonas) was isolated from the roots of upland cotton cultivar Zhongmian 44, and the FLiS gene was cloned. The FLiS protein purified in vitro can induce a hypersensitive response (HR) in tobacco, indicating that it is an active protein. In addition, it is capable of triggering an immune response in upland cotton, thereby enhancing the resistance to Verticillium wilt. The FLiS protein can induce the production of hydrogen peroxide(H2O2), callose, and defense enzymes in cotton, as well as the expression of disease resistance genes in the signaling pathways of salicylic acid (SA) and jasmonic acid (JA). FLiS can be used as a biological regulator to improve the resistance of upland cotton to *V. dahliae*.

## Highlights

- FLiS induces the occurrence of hypersensitive responses in tobacco and the production of immune substances in cotton.
- FLiS induces the expression of key disease resistance genes in the signaling pathways of SA and JA.
- FLiS can be applied as a biological regulator in the prevention and control of Verticillium wilt in cotton.

## 1 Introduction

Cotton is an important cash crop worldwide, and is a significant source of fiber, feed, foodstuff, oil and biofuel products (Gao et al., 2011). Verticillium wilt is the most destructive disease in cotton-growing areas, which prominently reduces cotton yield and fiber quality (Wang et al., 2016). Plants can enhance their disease resistance by improving their own immune responses. Plants mainly resist the infection of pathogenic bacteria through defense pathways such as pathogen-associated molecular pattern-triggered immunity (PTI) and pathogen-secreted effector-triggered immunity (ETI) (Naveed et al., 2020; Thomma et al., 2011; Zhou et al., 2022). The plant immune response first occurs through the mutual recognition between the recognition receptors of plant cells and the elicitors secreted by pathogenic bacteria, and then stimulates the systemic disease resistance of plants through a series of activated immune response signals (Nürnberger and Brunner, 2002; Bouizgarne et al., 2006; Jones and Dangl, 2006). The generation of the plant immune response is accompanied by the accumulation of disease-resistant substances such as lignin, callose and reactive oxygen species (ROS) (Marcec et al., 2019; Zhou et al., 2022; Sang and Macho, 2017). Elicitors can induce immune responses in plants. For example, the flagellin elicitors isolated from bacteria can induce immune responses in plants (Ma et al., 2017; Zhou et al., 2022; Wang et al., 2013). Flagellin is a keystone of pattern-triggered immunity in plants (Kalachova et al., 2022). As one of the Microbe-Associated Molecular Patterns, flagellin is a potent elicitor of hypersensitive cell death in plant cells (Taguchi et al., 2003). It induces the expression of numerous defense-related genes and triggers resistance to pathogenic bacteria (Zipfel et al., 2004). The FlgL secreted by the *B. velezensis* LJ02 activates the SA and ET signalling immune pathway, thereby improving plants disease resistance to TMV (Wei et al., 2021). Flagellin from *Brevibacllus Brevis* has proved to have the antibacterial activity (Wang et al., 2021). The interaction between calcium and reactive oxygen may be a critical element of their roles in information processing for plant defense responses (Marcec et al., 2019). Some studies have reports flagellin S (FLiS) can affect the motility of bacteria and also plays an important role in the synthesis of other flagellin, such as FlgM (Galeva et al., 2014). Flagellin induces plants to produce defense responses, which are accompanied by the accumulation of ethylene (ETH), callose, and reactive oxygen species (ROS), as well as the expression of disease resistance genes (Asai et al., 2002; Marcec et al., 2019). There are still few studies on the mechanism by which FLiS induces an immune response in cotton plants. In this study, the *FLiS* gene was cloned from the genome of *Pseudomonas*, and the FLiS protein was purified *in vitro*. As reported previously (Zhou et al., 2022), tobacco serves as a reliable bioassay system for evaluating the biological activity of purified proteins. In the present study, upon injection of the *in vitro*-purified FLiS protein into tobacco leaves, conspicuous cell necrosis was observed. This phenotypic response strongly suggests that the purified FLiS protein exhibits biological activity. FLiS may enhance the resistance of cotton to Verticillium wilt through the signaling pathways of salicylic acid (SA) and jasmonic acid (JA). Therefore, this study demonstrates for the first time the role and mechanism by which FLiS induces an immune response in upland cotton.

## 2 Materials and methods

### 2.1 The growth and preservation of *V. dahliae*

*V. dahliae* was generously provided by the College of Plant Protection, Huazhong Agricultural University. *V. dahliae* was grown on potato dextrose agar (PDA) medium and stored in a refrigerator at 4°C (Hu, 2012; Zhou et al., 2022).

### 2.2 Plant materials and culture conditions

The seeds of upland cotton cultivar Jimian 11 and *Nicotiana benthamiana* were sown in nutrient pots containing nutrient soil and vermiculite (3:1) and then transferred to a greenhouse for cultivation. The cultivation conditions were as follows: a photoperiod of 16 hours of light and 8 hours of darkness, a temperature of 28°C during the light period, a temperature of 23°C during the dark period, and a relative humidity of 60% (Zhou et al., 2022).

### 2.3 Construction, transformation, expression and purification of the FLiS expression vector

The Pet-28a expression vector, procured from Beijing Kinco Xinye Biotechnology Co., Ltd., was employed for the prokaryotic expression and purification of the FLiS protein. A set of specific primers (Pet-28a-FLiS-F/Pet-28a- FLiS-R) was meticulously designed based on the full-length open reading frame (ORF) sequence of the *FLiS* gene. Initially, 100 μL of the competent Escherichia coli cell suspension was retrieved from the -80°C freezer and thawed on ice. Subsequently, 1 μg of the recombinant expression plasmid Pet-28a-FLiS was added, followed by gentle mixing. The mixture was then incubated on ice for 30 minutes. A heat-shock treatment was performed in a 42°C water bath for 60 seconds, immediately followed by rapid cooling on ice for 5 minutes. Next, 1 mL of LB liquid medium (antibiotic- free) was added to the tube, and the contents were gently pipetted to ensure thorough mixing. The tube was then placed on a shaker at 37°C and 220 rpm for 1 h to allow the bacteria to resume normal growth. After centrifugation at 5000 rpm for 4 minutes, a portion of the supernatant was removed, leaving 150 μL of the medium. The bacterial cells were gently resuspended by pipetting and then spread onto an LB plate containing antibiotics. The plate was left upright until the bacterial solution was fully absorbed by the medium, after which the petri dish was inverted, sealed with parafilm, and incubated at 37°C overnight to obtain transformed single colonies. Following a positive result from the bacterial solution PCR of the transformed competent Escherichia coli BL21 (DE3) cells, prokaryotic expression was initiated. To assess the purifiability of the FliS protein, a single colony of the positive strain was selected and inoculated into 5 mL of fresh LB liquid medium supplemented with 50 mg/L ampicillin, followed by shaking culture at 37°C. When the optical density at 600 nm (OD600) reached 0.8, isopropyl-β-D-thiogalactopyranoside (IPTG) was added to a final concentration of 0.6 mM. The culture was then incubated on a shaker at 28°C and 160 rpm for 6 hours. The FLiS protein was purified *in vitro* using the Beyotime His-tagged Protein Purification Kit (Shanghai, China). The purification of the soluble FLiS protein was verified by sodium dodecyl sulfate–polyacrylamide gel electrophoresis (SDS–PAGE, 12.5%).

### 2.4 Detection of the biological activity of FLiS protein

The leaves of Nicotiana benthamiana at the four-leaf stage were used as experimental materials. 50 μL of the FLiS protein solution (with a concentration of 100 μg/mL) was injected from the abaxial epidermis of the tobacco leaves into the mesophyll, and water was used as a control for injection (Zhou et al., 2022). Three leaves were injected simultaneously in both the treatment group and the control group.

### 2.5 Methods for investigating the disease severity index of cotton

Cotton seedlings at the two-leaf stage with uniform growth were selected. The concentration of FLiS protein was adjusted to 100 μg/mL and sprayed evenly onto the leaves of upland cotton until the solution started to drip. Meanwhile, water was sprayed as a control. Two days after the application of the FLiS protein solution, the concentration of the *V. dahliae* spore suspension was adjusted to 1×10^7^ CFU/mL. The upland cotton seedlings were removed from the nutrient pots. The roots of the plants in the treatment group were soaked in the pathogen spore suspension for 30 minutes, while those in the control group were soaked in water for the same duration. Three replicates were set up for both the treatment group (treated with FLiS protein) and the control group (treated with water). Each replicate consisted of 36 cotton seedlings. Both the treatment and control groups were inoculated with *V. dahliae*. Fifteen days after inoculation with the spores of *V. dahliae*, the infection caused by *V. dahliae* in the roots, stems, and leaves of six randomly selected cotton plants from each replication was observed under a microscope (MVX10 MacroView microscope, Olympus, Japan). Thirty days after pathogen inoculation, the disease severity of the remaining 30 seedlings was scored according to the following criteria: 0 = healthy, no symptoms on the leaves; 1 = one or two cotyledons showing symptoms; 2 = a single true leaf showing symptoms; 3 = more than two leaves showing symptoms; 4 = plant death. The calculation methods for the overall disease index and control efficacy are as follows (Liu et al., 2014; Zhou et al., 2022).

### 2.6 Detection of H_2_O_2_ and callose contents in cotton leaves

The accumulation of reactive oxygen species (ROS) in cotton leaf cells induced by the protein FLiS was detected using the 3,3′-diaminobenzidine (DAB) tissue staining method. Three replicates were set up for both the treatment group (treated with FLiS protein) and the control group (treated with water). Each replicate consisted of 3 cotton seedlings. Cotton plants leaves were carefully excised 2 days after treatment, rinsed thoroughly with distilled water, and then placed in a petri dish. An appropriate volume of 1 mg/mL DAB staining solution (pH = 3.8) was added, and the leaves were stained under light at 25°C for 8h. After staining, the DAB solution was removed, and the leaves were immersed in a 95% ethanol solution to remove chlorophyll. Subsequently, the leaves were subjected to a boiling water bath for 15 min. The liquid in the petri dish was discarded, and absolute ethanol was added again followed by another boiling water bath. This process was repeated until the green color of the leaves was completely removed. Finally, the leaves were suspended in clean water. The leaves were then mounted on glass slides and photographed under a microscope for observation. The determination of callose content followed the method described by Millet et al. (2010). Cotton leaves were collected 2 days after treatment and fixed in a fixative solution of ethanol: acetic acid (3:1) for 3h to remove chlorophyll. After fixation, the leaves were taken out and dehydrated in 70% ethanol for 2h, then rehydrated in 50% ethanol for 2h, and finally soaked in water overnight. The leaves were gently rinsed with water 3 times and then incubated in a 10% NaOH solution at a constant temperature of 37°C for 2 h to render them transparent. After being rinsed gently with water 4 times, the leaves were incubated in 0.01% aniline blue in the dark for 4 h. After staining, the leaves were placed on glass slides, and the callose content was observed under a fluorescent stereomicroscope using ultraviolet excitation light.

### 2.7 Detection of defense enzymes activity and lignin content

Three replicates were set up for both the treatment group (treated with FLiS protein) and the control group (treated with water). Each replicate consisted of 30 cotton seedlings. Forty-eight hours after the cotton plants were inoculated with *V. dahliae*, The Leaves of each group were selected to detect different enzymes activities and lignin content. The method for determining superoxide dismutase (SOD) activity was referred to Zhang et al (2023). Two grams of wheat leaves from the same position were taken and ground into powder in liquid nitrogen. An appropriate amount of 50 mmol/L phosphate buffer solution (pH 7.5) was added. After thorough mixing, the mixture was extracted at 4°C for 24 h and then filtered. The filtrate was centrifuged at 8000 r/min for 20 min, and the supernatant was used as the crude SOD enzyme solution. At 25°C, a certain amount of the enzyme solution to be tested was added to 4.5 mL of 50 mmol/L Tris-HCl buffer solution (pH 8.2), and the mixture was pre-heated for 20 min. Then, 10 μL of 50 mmol/L pyrogallol was added, and the solution was quickly shaken well and poured into a cuvette. Using the Tris-HCl buffer solution as a blank control, the absorbance was measured at a wavelength of 325 nm every 30 s for a total of 6 times. Under these conditions, one unit of SOD activity was defined as the amount of enzyme that inhibited the auto-oxidation rate of pyrogallol by 50% per minute. The method for determining phenylalanine ammonia-lyase (PAL) activity was referred to Chen et al. (2009). 0.2 g of leaf samples that had been ground uniformly in liquid nitrogen were added to 1 mL of borate buffer (pH 8.8, containing 5 mM 2- mercaptoethanol and 1% PVP). After shaking, the mixture was placed at 4°C and then centrifuged at 8000 g for 20 min. 200 μL of the enzyme extract was added to 800 μL of the reaction solution, and the mixture was incubated in a 30°C water bath for 30 min. Then, 100 μL of 5 M HCl was added to terminate the reaction, and the absorbance at 290 nm was measured. The method for determining catalase (CAT) activity was referred to Zhang et al (2023). 0.2 g of leaf samples ground uniformly in liquid nitrogen were added to 1 mL of 0.1 M phosphate buffer (pH 7.0). After shaking to mix well, the mixture was centrifuged at 12000 rpm at 4°C for 15 min. 100 μL of the supernatant enzyme solution was added to 100 μL of 2% H_2_O_2_ and 800 μL of 0.1 M phosphate buffer (pH 7.0). The kinetic curve at 240 nm was measured for 1 min. The method for determining peroxidase (POD) activity was referred toZhang et al (2023). 0.2 g of leaf samples ground uniformly in liquid nitrogen were added to 1 mL of 0.1 M phosphate buffer (pH 7.0). After shaking to mix well, the mixture was centrifuged at 12000 rpm at 4°C for 15 min. 100 μL of the supernatant enzyme solution was added to 100 μL of 0.1 M catechol, 50 μL of 2% H_2_O_2_, and 800 μL of 0.1 M phosphate buffer (pH 7.0). The kinetic curve at 470 nm was measured for 1 min. The method for determining polyphenol oxidase (PPO) activity was referred to Richter et al. (2012). 0.2 g of leaf samples ground uniformly in liquid nitrogen were added to 1 mL of 0.05 mol/L phosphate buffer (pH 5.5). After shaking to mix well, the mixture was centrifuged at 12000 rpm at 4°C for 15 min. 0.5 mL of the supernatant enzyme solution was added to 1.0 mL of 0.1 M catechol solution and 1.5 mL of 0.05 mol/L phosphate buffer (pH 5.5), with a total volume of 3 mL. Immediately after thorough mixing, the absorbance was measured at 398 nm every 2 min. Using 0.5 mL of 0.05 mol/L phosphate buffer (pH 5.5) as a control. The method for determining lignin content was referred to Xiong et al (2021). The lignin content was measured using a kit produced by Ruiyuan Biotechnology Co., Ltd. (Shanghai). The wheat stalks were dried and ground into powder, and then passed through an 80-mesh sieve. 3 mg of the sample was weighed and placed in a 2.5 mL centrifuge tube, and 500 μL of sulfuric acid and 20 μL of perchloric acid were added. The tube was sealed with a sealing film, mixed well, and shaken every 10 min in an 80°C water bath for 40 min, and then cooled to room temperature. 0.5 mL of the sample was transferred to a 50 mL centrifuge tube, and 995 μL of NaOH solution was added and mixed well. 200 μL of each sample solution was placed in a 96-well plate. The absorbance values of the blank tube and the measurement tube were measured at 280 nm using a microplate reader.

### 2.8 Statistical analysis

All the experiments in this study were repeated at least three times. The experimental data obtained from the experiments were subjected to analysis of variance (ANOVA), Student’s t-test, and one-way ANOVA (P < 0.05) using SPSS 19.0 software (SPSS Inc.). Subsequently, Duncan’s test was performed for multiple comparisons.

## 3 Results

### 3.1 Prokaryotic expression, purification of FLiS protein and detection of its biological activity

**Figure 1:**
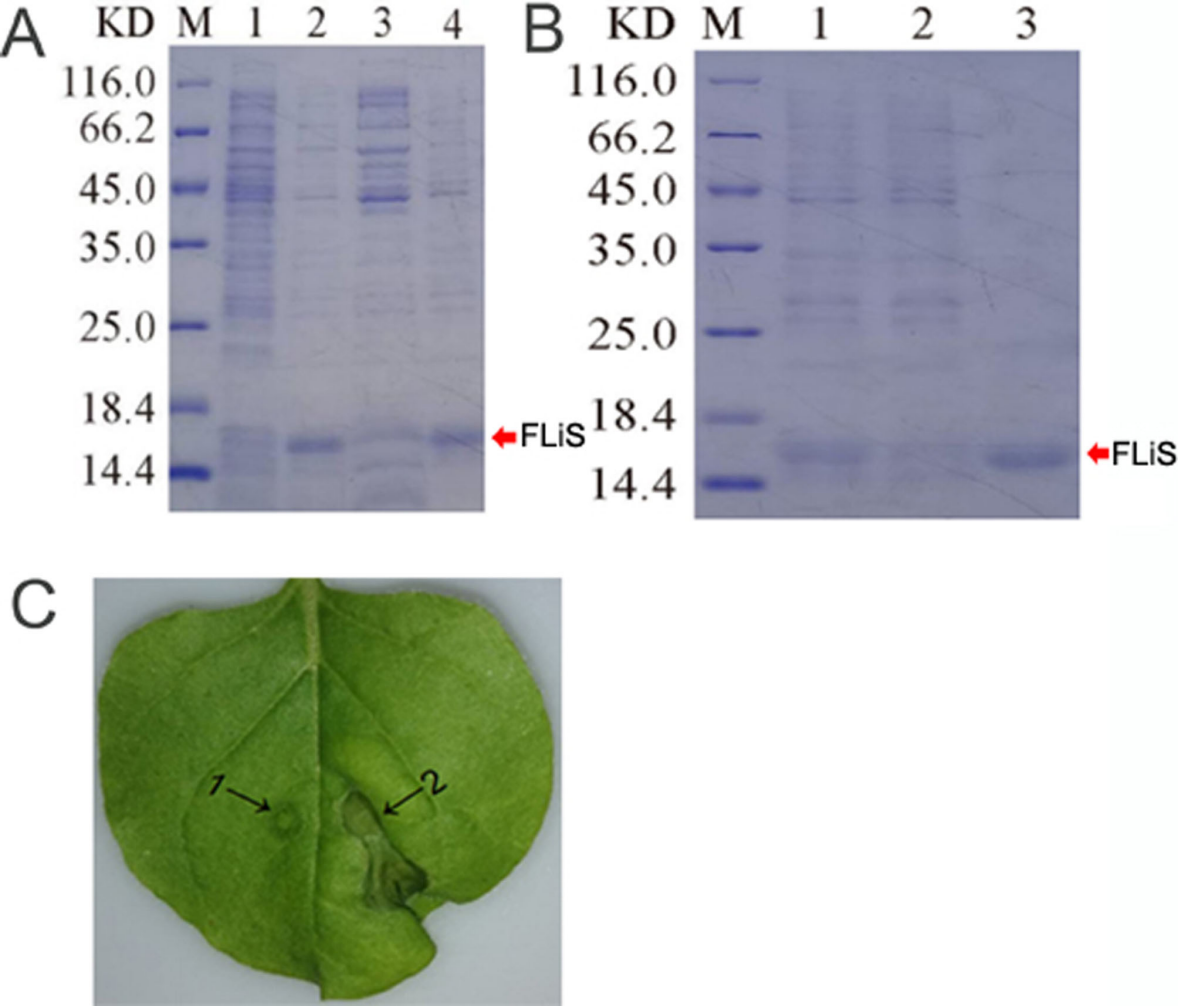
The purified FLiS protein induces a hypersensitive response in tobacco leaves. **(A)** (M: protein molecular mass standard, 1: not induced, 2: induced, 3: induced crushing supernatant, 4: induced crushing precipitation), **(B)** (M: protein molecular mass standard,1: processed sample after crushing,2: effluent,3: elution), **(C)**, determination of bioactivity of FLiS recombinant protein (1: Control, 2: FLiS).

The FLiS gene was cloned from an endophytic bacterium (*Pseudomonas aeruginosa*) isolated from the roots of the upland cotton cultivar Zhongmian 44. To verify whether the FLiS protein with biological activity could be obtained through prokaryotic expression and purification *in vitro*, the Pet-28a-FLiS recombinant plasmid was transformed into the Escherichia coli strain BL21 (DE3). After being cultured at 37°C until the OD600 value reached 0.8, it was induced with 0.6 mmol of isopropyl-β-D-thiogalactoside (IPTG) at 28°C for 6 hours. The size of the purified FLiS protein in the supernatant, as examined by SDS-PAGE, was 15.14 kilodaltons (kDa) (**Figures 1A, B**). To further verify the activity of this protein, the FLiS protein was injected into tobacco, and it was found that it induced a hypersensitive response (HR) effect in tobacco leaves (**Figure 1C**). The above results indicate that the FLiS protein obtained through prokaryotic expression and purification possesses biological activity.

### 3.2 FLiS protein reduces the disease index of cotton

**Figure 2:**
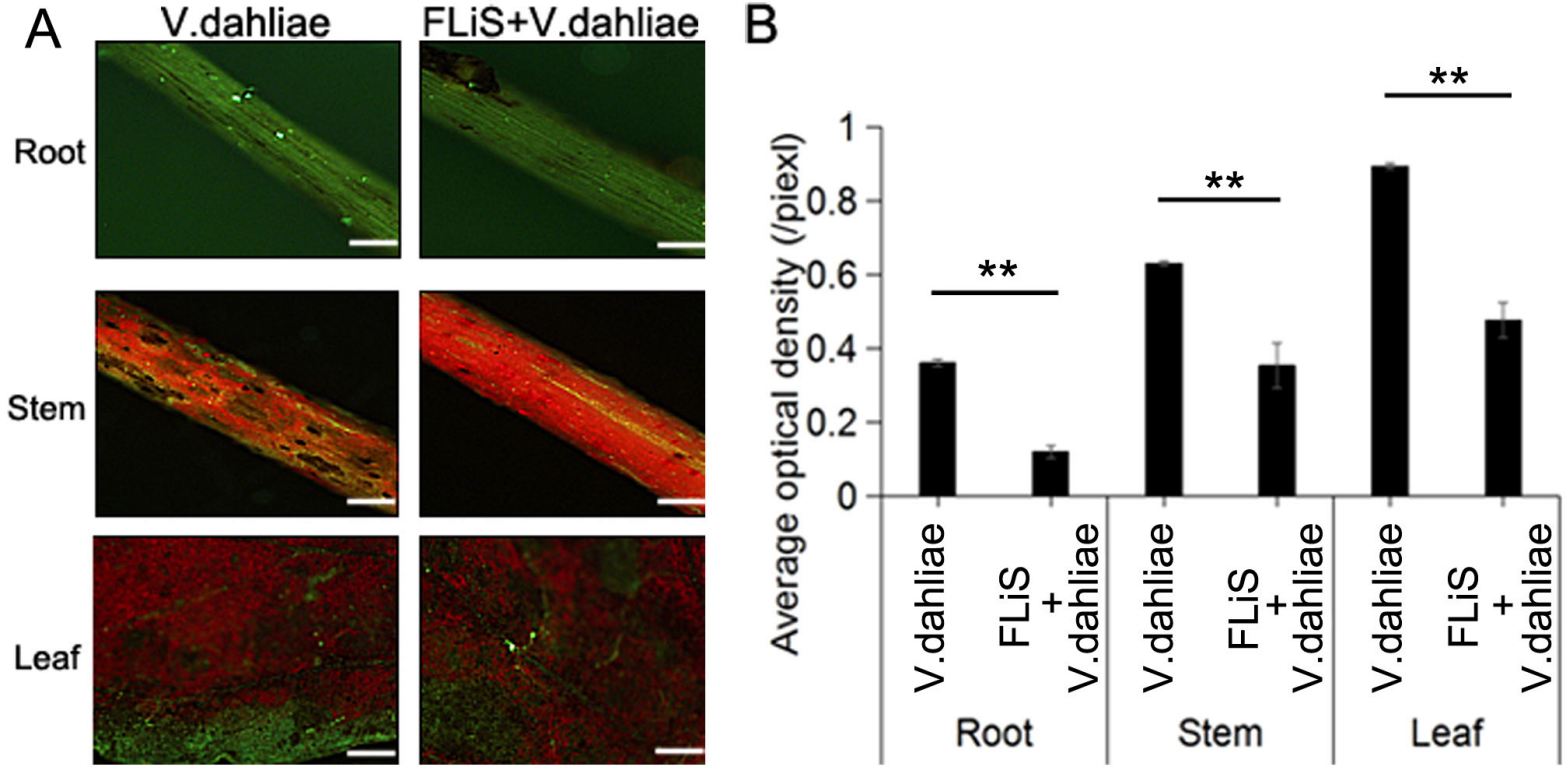
FLiS protein reduces the disease index of cotton **(A)** Spore colonization in the roots, stems, and leaves of cotton plants. **(B)** Quantification of the average fluorescence density of spores in the roots, stems, and leaves of cotton plants. Data are presented as means ± standard deviations (SDs), with n = 3 biological replicates. Significance was determined using a t-test (**, P < 0.01). Scale bars = 500 µm.

Cotton seedlings at the two-leaf stage with uniform growth were selected. The concentration of FLiS protein was adjusted to 100 μg/mL and evenly sprayed onto the cotton leaves using a spray bottle until the liquid started to drip. Water was used as the control. Two days after the treatment with the FLiS solution, the concentration of the *V. dahliae* pathogen spore suspension was adjusted to 1×10^7^CFU/mL, and the seedlings were inoculated with it. Under the microscope, it was observed that the amount of *V. dahliae* infection in the roots, stems, and leaves of the plants sprayed with the FLiS protein solution was significantly lower than that in the control group. This indicates that the FLiS protein can enhance the resistance of cotton to *V. dahliae* (**Figures 2A, B**). Thirty days after the treatment with the FLiS protein, the disease index of the treatment group was significantly lower than that of the control group (**Table 1**). The relative biocontrol efficacy of FLiS against the disease in cotton plants was 12.33 (**Table 1**). The above results demonstrate that the FLiS protein can improve the resistance of cotton to verticillium wilt.

**Table 1:** The control effect of FLiS protein on cotton.

Treatment	Disease index (DI)	Relative control effect (%)
Jimian 11 (Control)	75.00 ± 3.61a	
Jimian 11 (FLiS)	65.75 ± 3.61b	12.33 ± 4.81

Control: Treated with H_2_O; FLiS: Treated with FLiS protein. Significance was determined using a t-test. Significance was determined at the P < 0.05 level, and different letters denote significant differences among groups.

### 3.3 FLiS induced H_2_O_2_ accumulation in cotton leaves

**Figure 3:**
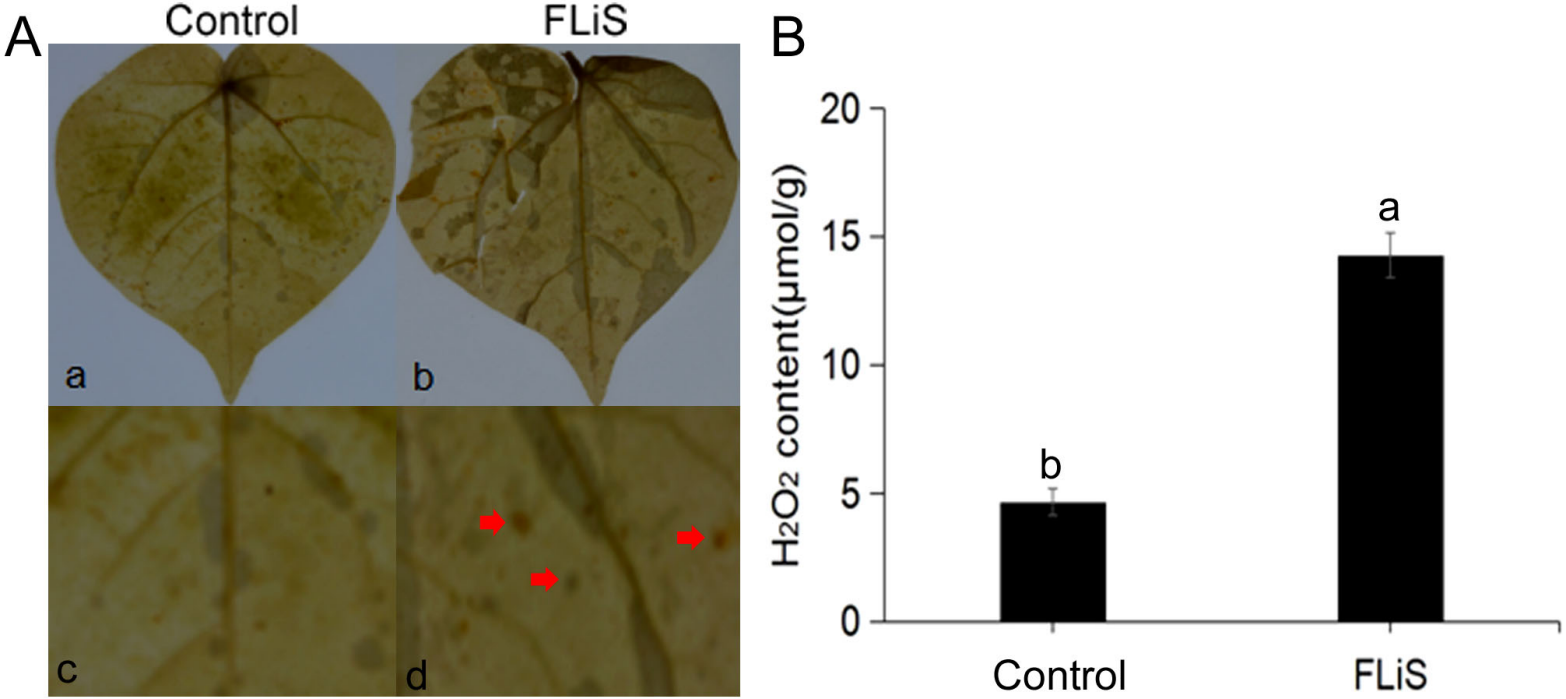
FLiS protein induces the production of hydrogen peroxide in cotton leaves **(A)** Induction of hydrogen peroxide deposition in cotton leaves by FLiS(a:Control(H_2_O); b,FLiS; c: magnified image of the CK group; d: magnified image of the FLiS group). The concentration of FLiS was 100 µg/mL, and cotton leaves were treated for 48 (h) **(B)** Quantification of hydrogen peroxide content in cotton leaves. Data are presented as means ± standard deviations (SDs) based on three independent biological replicates (n = 3). Significance was determined using a t-test. Significance was determined at the P < 0.05 level, and different letters denote significant differences among groups.

The diaminobenzidine (DAB) tissue staining method was employed to detect the generation and accumulation of hydrogen peroxide (H_2_O_2_) molecules in plant leaves. In order to explore whether FLiS protein can induce H_2_O_2_ production, the DAB staining results of cotton leaves treated with FLiS protein for 48h showed that H_2_O_2_ could be significantly detected in cotton leaves after FLiS protein treatment for 48h compared with the control group. The content of H_2_O_2_ in FLiS treatment group was significantly higher than that in control group (**Figures 3A, B**). The results showed that FLiS protein could induce the outbreak of H_2_O_2_ in leaves to induce the defense response of cotton.

### 3.4 FLiS induces the deposition of callose in cotton leaves

**Figure 4:**
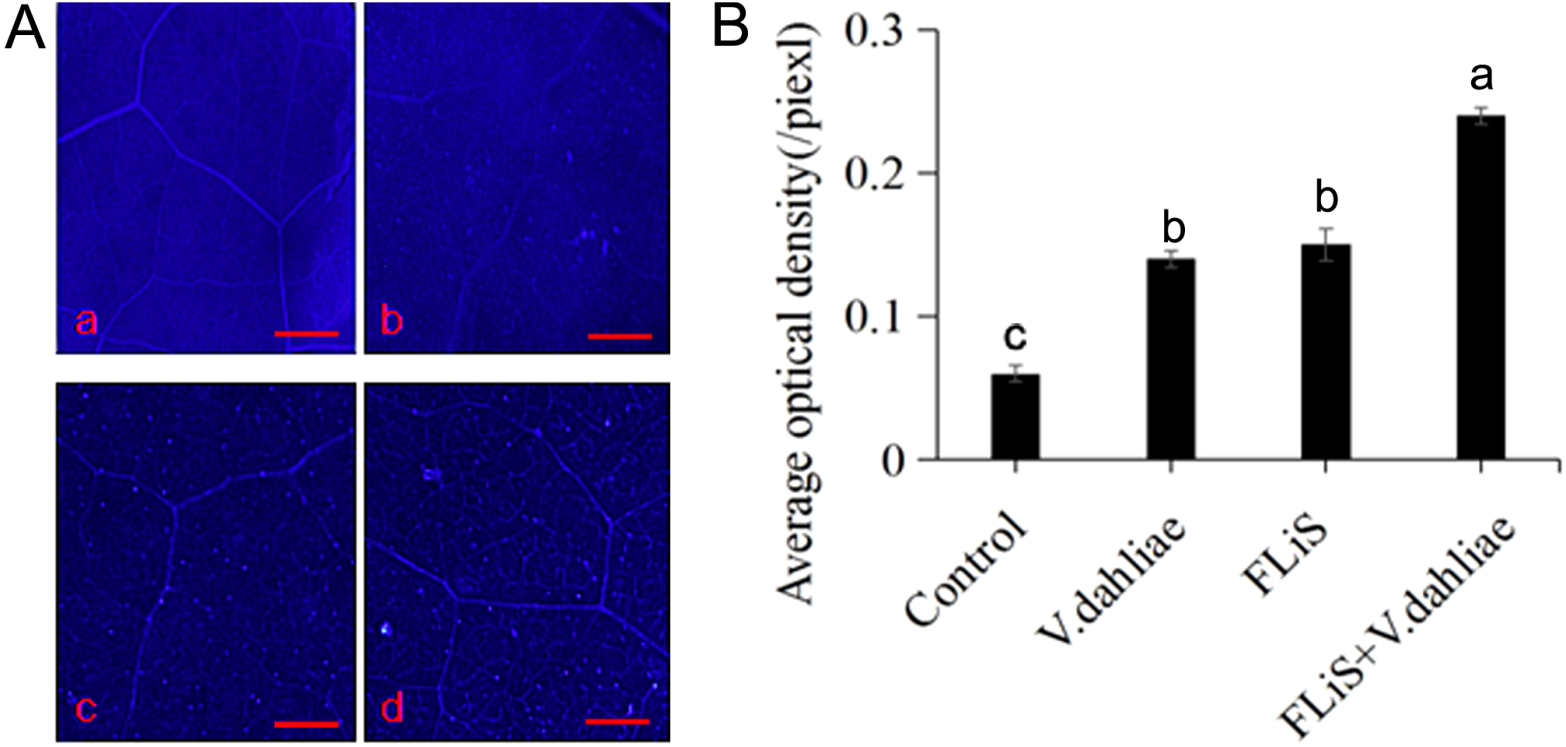
FLiS protein induces the production of callose in cotton leaves **(A)** Induction of callose deposition in cotton leaves by FLiS(a:Control(H_2_O); b:*V. dahliae*; c,FLiS; d:FLiS+*V. dahliae*). The concentration of FLiS was 100 µg/mL, and cotton leaves were treated for 48 (h) Callose was visualized, and the scale bar represents 1000 µm. **(B)** Quantification of the average optical density of callose in cotton leaves. Data are presented as means ± standard deviations (SDs) based on three independent biological replicates (n = 3). Statistical analysis was performed using one-way analysis of variance (ANOVA) followed by Duncan’s multiple range test. Significance was determined at the P < 0.05 level, and different letters denote significant differences among groups.

The accumulation of callose is a common disease-resistant substance in the process of plant immunity. To determine whether the FLiS protein can induce the production of callose in cotton leaves, cotton leaves were sprayed with the FLiS protein solution. Forty - eight hours after the treatment, obvious callose deposits were observed in the FLiS-treated leaves compared with the control group (**Figures 4A, B**). These results indicate that FLiS can induce an immune response in cotton, accompanied by the accumulation of callose, thereby enhancing the resistance of cotton to *V. dahliae*.

### 3.5 FLiS induces an increase in the activity of defensive enzymes and the content of lignin

**Figure 5:**
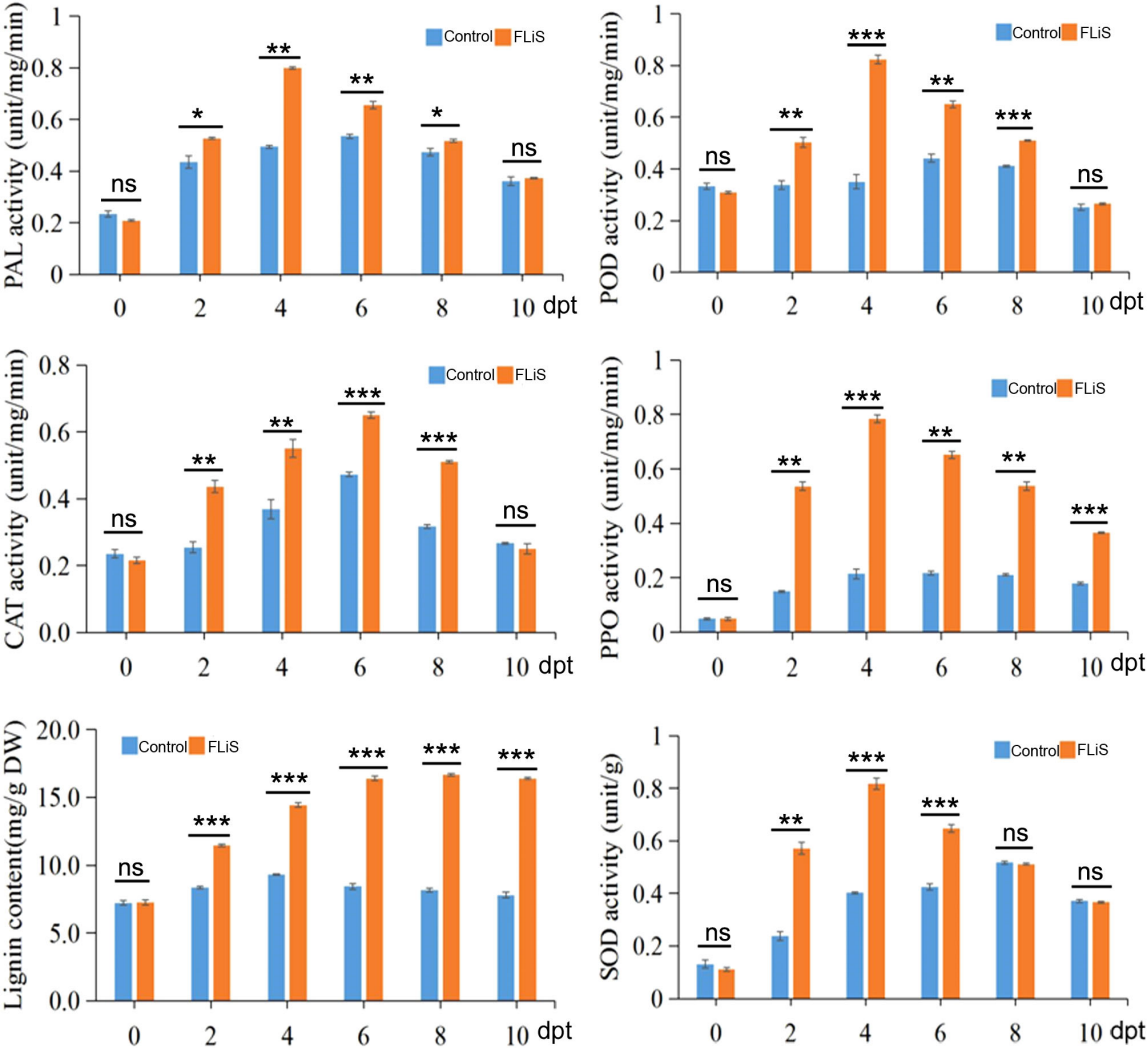
FLiS induces an increase in the activity of defensive enzymes and the content of lignin Note: After the plants were sprayed with the FLiS solution, the activities of the five defense-related enzymes(superoxide dismutase (SOD), catalase (CAT), polyphenol oxidase (PPO), peroxidase (POD) and phenylalanine ammonia-lyase (PAL)) and lignin content were checked. Control: Treated with H_2_O; FLiS: Treated with FLiS protein. *: significant at 0.05 level, **: significant at 0.01 level,***: significant at 0.001 level, ns, no significance.

To further verify whether the activity of defensive enzymes increases while the FLiS protein induces an immune response in cotton, we detected the changes in the activities of five enzymes and the lignin content in the cotton leaves sprayed with the FLiS solution. After the plants were sprayed with the FLiS solution, the activities of the five defense-related enzymes increased to varying degrees (**Figure 5**). Specifically, the activities of superoxide dismutase (SOD), catalase (CAT), polyphenol oxidase (PPO), peroxidase (POD), and phenylalanine ammonia-lyase (PAL) were significantly higher than those of the control group (**Figure 5**). In addition, the lignin content was also significantly higher than that of the control group. These results indicate that during the process of the FLiS protein inducing an immune response in cotton, the increase in the lignin content and the activities of defense-related enzymes in cotton leaves enhances the resistance of cotton to *V. dahliae*.

### 3.6 FLiS protein induces expression of relative resistance genes

**Figure 6:**
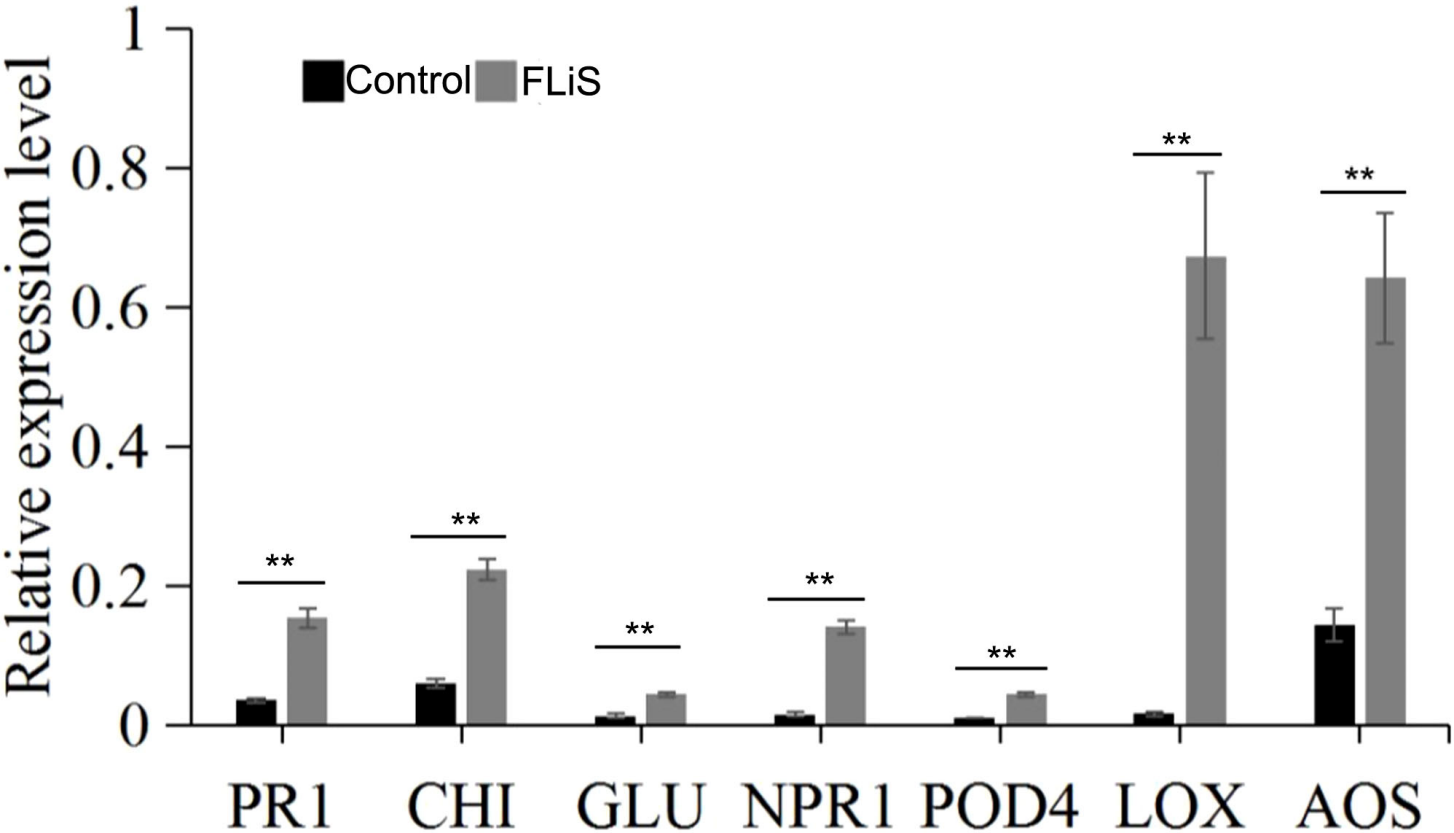
FLiS protein induces the expression of defense-related genes in cotton leaves Note: After the plants were sprayed with the FLiS solution, the expression level of defense-related genes (pathogenesis-related protein 1(PR1), β-1, 3-glucanase (GLU), chitinase (CHI), pathogenesis-related gene 1(NPR1), peroxidase 4 (POD4), lipoxygenase (LOX), allene oxide synthase (AOS)) were checked. Control: Treated with H_2_O; FLiS: Treated with FLiS protein. **: significant at 0.01 level.

In the process of plant induced resistance, the improvement of resistance is always accompanied by the up-regulation of defense-related genes, which mainly include pathogenesis-related protein 1(PR1), β-1, 3-glucanase (*GLU*), chitinase (*CHI*) and other genes (Zhang et al., 2008; Cheng et al., 2005). Forty-eight hours after spraying the FLiS protein, the gene expressions of *PR1*,*CHI* and *GLU* in Jimian 11 were significantly increased compared with the control (**Figure 6**). The results showed that FLiS protein could induce cotton plant to produce defense response against pathogen infection.

Jasmonic acid/ethylene (JA/Et) signaling pathway and salicylic acid (SA) signaling pathway are two important signaling pathways in plant defense response system (Turner et al., 2002; Pieterse et al., 2009). After spraying FLiS protein for 48 hours, the relative expression levels of key genes of salicylic acid signaling pathway Nonexpressor of pathogenesis-related gene 1(*NPR1*), peroxidase 4 (*POD4*), key genes of jasmonic acid pathway lipoxygenase (*LOX*) and allene oxide synthase (*AOS*) were detected respectively (**Figure 6**). The results showed that the relative expression levels of two key genes of SA pathway, *NPR1* and *POD4*, were higher than those in control. The relative expression levels of two key genes of JA pathway, *LOX* and *AOS*, were higher than those in control (**Figure 6**). Therefore, FLiS protein may induce cotton defense response mainly depends on JA and SA signaling pathways.

## 4 Discussion

### 4.1 FLiS protein structure

In this study, the full-length flagellin gene FLiS was cloned from the whole genome DNA of endophytic *Pseudomonas* of cotton. Bioinformatics analysis showed that the amino acid sequence corresponding to FLiS gene was not very similar to that of Flg22, which may be a new flagellin gene Flg22 is a conserved polypeptide fragment of the N-terminal of flagellin, containing 22 amino groups (Gómez-Gómez and Boller, 2000). FliS is the cytoplasmic flagellate companion protein of flagellin, which polymerizes into filaments outside of flagellin (Lee et al., 2017). The cytoplasmic interaction between FliS and flagellin is essential for the retention of flagellin in monomer form, which is transported from the cytoplasm to the extracellular space for fiber assembly via flagellate output devices (Lee et al., 2017). The deficiency of FliS protein directly reduces the viability, pathogenicity and vitality of bacteria (Qi, 2015). FliS has a highly dynamic n-terminal region, which is attached to the common four-helical beam structure (Lee et al., 2017; Qi, 2015). An invariant proline residue is found in all known FliS sequences between the N-terminal region and the quad helix bundle (Lee et al., 2017; Qi, 2015). N-terminal proline residues play an important role in FliS dimerization and flagellin recognition (Lee et al., 2017).

### 4.2 Flagellin FLiS induces resistance

In this study, Jimian 11 was used as the research material to explore the possible resistance pathways of cotton to *V. dahliae* induced by the protein FLiS. By studying a series of defense responses induced by recombinant protein FLiS, it was found that similar to the early response triggered by Flg22, FLiS protein may induce the production of defense signals H_2_O_2_ and callose in cotton cells. These related substances produced in early defense responses are key small chemical molecules in the FLiS-induced resistance signaling pathway in cotton plants, regulating the plant’s immune system.

PAL, PPO and POD are three enzymes involved in plant disease resistance metabolism. In this experiment, the increase of PAL activity can strengthen the phenylpropanoid metabolic pathway and play a chemical barrier role in plant disease resistance. Therefore, the activity level of phenylalanine ammonlyase can be used as a biochemical index of plant disease resistance (Klarzynski et al., 2000, Klarzynski et al., 2003). PPO is widely present in plants and can oxidize phenolic substances into quinones or their derivatives to enhance the host’s resistance to pathogens (Mandal and Mitra, 2007). In this experiment, the increase of PPO activity led to the increase of phenol oxide in cotton seedling leaves, and phenol oxide had inhibitory or toxic effects on many pathogens. Therefore, it may be one of the mechanisms of cotton seedling resistance to verticillium wilt. POD is an important defense enzyme in plants. The activity of this enzyme was positively correlated with plant resistance (Mohan and Kolattukudy, 1990; Mclusky et al., 1999), this enzyme can synthesize cell wall precursor substances and thicken cell wall to form a barrier against pathogen invasion. SOD and CAT enzyme activity also are important defense enzyme in plants (Zhang et al, 2023). After treatment with FLiS protein, the enzyme activities of PAL, PPO, POD, SOD and CAT were increased to varying degrees, suggesting that the biochemical mechanism of systemic resistance induced by FLiS protein may be due to the improvement of the activity of disease-resistant enzymes. However, this experiment only used cotton susceptible variety (Jimian 11) as test materials. Whether the test results and related conclusions can be applied to other cotton varieties needs further verification. In addition, leaves were used as materials to study the activities of various protective enzymes, and the correlation between changes in other parts of cotton and resistance to verticillium wilt is still needed to be studied. Flg22 can induce defense response in *Arabidopsis thaliana*, such as burst of reactive oxygen species, deposition of callose and expression of defense genes *PR1*, pathogenesis - related protein 5 (*PR5*), phenylalanine ammonia - lyase 1(*PAL1*) and Glutathione S - transferase 1(*GST1*) (Zipfel et al., 2004). In addition, Flg22 induces allergic reactions, reactive oxygen species outbreaks, and ethylene synthesis in tobacco and tomatoes (Felix et al., 1999). Flg22 can also induce allergic reactions in higher plants (Naito et al., 2008). However, the defense response induced by Flg22 in higher plants is mainly based on salicylic acid signaling, and is coordinated with jasmonic acid and ethylene signaling pathways (Thomma et al., 2011). In this study, compared with the control, FLiS protein pretreatment can effectively reduce the degree of verticillium wilt infection in cotton, indicating that The FLiS protein has the potential to enhance the resistance of cotton against *V. dahliae*. Some disease-course related genes, such as *PR1*, *CHI*, and *GLU*, are involved in the plant defense pathway when pathogens infect the plant (Shi et al., 2015). Plant defense response depends mainly on the presence of disease-course related genes and the state of expression of these genes (Chen et al., 2012). In this study, the expression level of *PR1*, *CHI*, and *GLU* in cotton treated with FLiS protein was analyzed. Pretreated with FLiS protein can induce the high level of expression of resistance genes, which explains the mechanism of FLiS protein inducing verticillium wilt in cotton at the molecular level. Similar results have been reported in other plants (Ahn et al., 2005). In this study, the relative expression levels of two key genes of SA pathway, *NPR1* and *POD4*, were significantly increased. the relative expression levels of two key genes of JA pathway, *LOX* and *AOS*, were significantly increased. Therefore, FLiS protein elicitor induced cotton defense response mainly depends on JA and SA pathways.

## 5 Conclusions

In this study, an endophytic bacterium (*Pseudomonas*) was isolated from the roots of the cotton variety Zhongmian 44 for the first time, and the *FLiS* gene was subsequently amplified. Our findings revealed that the FLiS protein triggered a hypersensitive response in tobacco leaves and elicited an immune response in upland cotton. Mechanistically, the FLiS protein is hypothesized to potentiate the immune response against *V. dahliae* infection by modulating the JA and SA signaling pathways. Collectively, these results suggest that FLiS holds promise as a bioregulator for augmenting the resistance of upland cotton to *V. dahliae*.

## Data Availability

The datasets presented in this study can be found in online repositories. The names of the repository/repositories and accession number(s) can be found in the article/**Supplementary Material**.
