# Supplementary material for: Flagellin FLiS improves the resistance of cotton to Verticillium wilt through the signaling pathways of salicylic acid and jasmonic acid
Source: Front Plant Sci. 2025 Jun 3;16:1595529. doi: 10.3389/fpls.2025.1595529 (PMC12199450; doi:10.3389/fpls.2025.1595529)
Supplement: Supplementary file 1 [file Table1.docx]

**Supplementary Table 1** Primer sets used for qRT–PCR

| Genes | Sequences |
| --- | --- |
| CHI-F | ACCAAGCTACTCGCAAGAGG |
| CHI-R | CGGAAGCGCAGTAAGATGA |
| GLU-F | CATTGATATGACCTTGATCG |
| GLU-R | GTGAGATATCCCTTGGATTG |
| PR1-F | TGCCCAAGACTCACAACAAG |
| PR1-R | GGCCTTCTCATTAACCCACA |
| POD4-F | TTTGCTGCTGCCATGGTGAAGATG |
| POD4-R | CAATCAGTTGACCACCCTGCAGTT |
| LOX-F | AGTCGTCGGTTCATGCCTGAGAAA |
| LOX-R | ATTCCCAGGAGTGTCTGCAGTTGA |
| NPR1-F | GCGCGGATCCATGGATAATAGTAGGACTGCGTT |
| NPR1-R | GCGCCTCGAGTTTCCTAAAAGGGAGCTTATTGGG |
| AOS-F | CGGACCTACACCGTTCATTAGC |
| AOS-R | CGTGACTTTCAATCAACAAATCCAC |
